# Skill execution and sleep deprivation: effects of acute caffeine or creatine supplementation - a randomized placebo-controlled trial

**DOI:** 10.1186/1550-2783-8-2

**Published:** 2011-02-16

**Authors:** Christian J Cook, Blair T Crewther, Liam P Kilduff, Scott Drawer, Chris M Gaviglio

**Affiliations:** 1 UK Sport Council, 40 Bernard St London, UK; 2 Sport and Exercise Science Research Centre, Swansea University, Swansea, UK; 3 Hamlyn Centre, Institute of Global Health Innovation, Imperial College, London, UK; 4 Department for Health, University of Bath, Bath, UK; 5 Queensland Academy of Sport and Gold Coast SUNS, AFL Franchise Gold Coast, Brisbane, Australia

## Abstract

**Background:**

We investigated the effects of sleep deprivation with or without acute supplementation of caffeine or creatine on the execution of a repeated rugby passing skill.

**Method:**

Ten elite rugby players completed 10 trials on a simple rugby passing skill test (20 repeats per trial), following a period of familiarisation. The players had between 7-9 h sleep on 5 of these trials and between 3-5 h sleep (deprivation) on the other 5. At a time of 1.5 h before each trial, they undertook administration of either: placebo tablets, 50 or 100 mg/kg creatine, 1 or 5 mg/kg caffeine. Saliva was collected before each trial and assayed for salivary free cortisol and testosterone.

**Results:**

Sleep deprivation with placebo application resulted in a significant fall in skill performance accuracy on both the dominant and non-dominant passing sides (p < 0.001). No fall in skill performance was seen with caffeine doses of 1 or 5 mg/kg, and the two doses were not significantly different in effect. Similarly, no deficit was seen with creatine administration at 50 or 100 mg/kg and the performance effects were not significantly different. Salivary testosterone was not affected by sleep deprivation, but trended higher with the 100 mg/kg creatine dose, compared to the placebo treatment (p = 0.067). Salivary cortisol was elevated (p = 0.001) with the 5 mg/kg dose of caffeine (vs. placebo).

**Conclusion:**

Acute sleep deprivation affects performance of a simple repeat skill in elite athletes and this was ameliorated by a single dose of either caffeine or creatine. Acute creatine use may help to alleviate decrements in skill performance in situations of sleep deprivation, such as transmeridian travel, and caffeine at low doses appears as efficacious as higher doses, at alleviating sleep deprivation deficits in athletes with a history of low caffeine use. Both options are without the side effects of higher dose caffeine use.

## Background

Both creatine and caffeine have found common use in sport [1-4] for a variety of training and competitive aims. Popular use of caffeine is often at high concentrations (4-9 mg/kg) on the basis that these are more efficacious, but the proof of this is low with individual variability and consumption habits being the more dominant factors [5,6]. In contrast, popular creatine supplementation dosages appear to have fallen as literature supports the contention that lower doses can be as effective as higher loading schemes, again individual variability and responsiveness being major determinants [4].

While the ability of acute caffeine to address cognitive related sleep deficits is reasonably established [7], it is only recently that creatine has demonstrated similar properties [8,9]. It has been suggested that sleep deprivation is associated with an acute reduction in high energy phosphates that in turn produces some degree of cognitive processing deficit [8-14]. Creatine supplementation has been shown to improve certain aspects of cognitive performance with sleep deprivation and to have some positive benefits in deficits associated with certain pathophysiologies [13,14]. If sleep deprivation is associated with an energy deficit then errors in performance are perhaps more likely to occur when concentration demands are high and/or for prolonged periods of repeated task execution. Some evidence suggests that it is tasks of this nature that are most affected by acute sleep deprivation [15].

Creatine has generally only been used in chronic loading protocols. However, if the contention that acute sleep deprivation reduces brain creatine is true, than an acute dose of creatine, as opposed to the classical longer loading periods, may alleviate some of these effects. This would be dependent on creatine uptake not being rate limited, something unknown for the brain. Creatine does however readily cross the blood brain barrier and chronic systemic loading does appear to increase brain stores [13,14]. Acute doses of caffeine appear most beneficial at around 30-90 min prior performance [16] and while the timing of an acute dose of creatine has yet to be determined, it appears to take at least an hour for absorption into the bloodstream [17-19].

Sleep deprivation is not uncommon around competition in sport particularly with the frequent demands of international travel. Assessing its effects on performance is however difficult, especially in team sports where multiple physical and skill components are involved. While overt physical components such as power don't appear affected by acute deprivation [20] a few studies do however suggest acute deprivation can affect certain sport skill and physical performance [21,22].

Given the potential usefulness of safe supplementation for alleviating cognitive deficits associated with sleep deprivation, this study aimed to investigate if acute administration of creatine or caffeine could offer this advantage. To this end, we tested the effects of acute occurring sleep deprivation on a fundamental rugby skill, passing the ball while running with accuracy, in elite level players. Further to this, we tested if acute administration of creatine or caffeine would in any way alter this performance.

## Method

### Subjects

Ten professional rugby backs (mean ± SD, age; 20 ± 0.5 years) that were in good health and injury-free volunteered for this trial. Subject bodyweights were 90 ± 4 kg and heights 1.81 ± 0.02 m (mean ± SD). Bodyweights showed no significant changes over the course of this trial. A within-treatment design was used with each subject acting as their own control to improve reliability and the sensitivity of measurements. Subjects all reported a low and infrequent history of both previous caffeine use (in any form) and each had used creatine previously, usually in a classic loading protocol. The athletes were all very low and infrequent social consumers of alcohol. A university ethics committee approved the study procedures and each subject signed an informed consent form before participation.

### Study design

A blinded, repeated measure, placebo-controlled crossover design was used to examine the effects of acute supplementation (caffeine or creatine) on the execution of a repeated rugby passing skill during sleep deprivation.

### Testing procedures

On days of testing the subjects consumed the same breakfast which consisted of a bowl of cereal with fruit, yoghurt and milk in a portion of voluntary choice and two poached eggs on one piece of buttered toast consumed between 0700 h and 0800 h. Water was available ad libitum. On the night previous to testing food was not strictly controlled but all subjects reported consuming a dinner of at least red meat and 3 vegetables and a latter evening protein milkshake.

Initially all 10 players in this study undertook 3 weeks of familiarisation training on a rugby-specific passing skill (total of 12 sessions). Changes in performance and variability were calculated over these sessions. Familiarisation was undertaken at 1130 h each time, and required 2 previous nights of greater than 7 h sleep to be performed (i.e. clearly non-sleep deprived). Following familiarisation the players were asked to keep a sleep log to record the number of hours slept per night. This was reported at 0900 h on Monday to Friday.

The skill testing procedures were performed on 10 separate occasions across a 10 week period (not less than three days apart) at 1130 h, with between 7-9 h sleep for two nights preceding five of these tests, and with 3- 5 h sleep (sleep deprived) on the night preceding (but more than 7 h on the previous night) on the other 5 trials. At 1000 h on the test days the athletes received one of the following: placebo tablets (sucrose at 5 mg/kg); creatine monohydrate tablets (50 or 100 mg/kg bodyweight); caffeine tablets (1 or 5 mg/kg bodyweight). Thus, the absolute mean dosages of creatine used were 4.5 g and 9 g, respectively, and caffeine dosages of 90 mg and 450 mg were respectively used. The doses were divided into 5 tablets, of same size based upon each individual athlete's bodyweight at the start of the trial, across all treatments. Maize starch was used where necessary to balance out tablet weights and tablets were hand made using gelatine capsules. Treatment (blinded) was randomised across athletes and the skill execution tests.

On all trials subjects refrained from alcohol consumption for at least 48 h prior to testing and from any caffeine and caffeine containing drinks for at least 24 h (athletes were infrequent caffeine drinkers). The athletes recruited had not used creatine or creatine-based supplements within the preceding 3 months of this study.

### Rugby passing skill test

The repeated rugby passing skill was performed indoors and consisted of: a 20 m sprint in which at the 10 m mark the player had to attempt to pass a rugby ball left or right (alternating) through a hanging hoop (diameter 1.5 m) 10 m away from them. Players were also asked to identify their better passing side (dominant). All 10 players clearly believed they had a better passing side, and this was supported by alternate accuracy. The 20 m protocol had to be completed in less than 20 s (beep timed for the players) and they undertook 20 repeats (10 passes on each side) with a walk back recovery period. Execution success was simply defined as the number of successful attempts on the dominant and non-dominant side. The elite group of athletes were familiar with this common rugby skill and thus, a high level of reliability was expected. To further ensure high test-retest reliability, three weeks of familiarization sessions were also performed before the main testing procedures.

### Saliva measures

Saliva was collected immediately before each trial as follows: players provided a passive drool of saliva into sterile containers (LabServe, NewZealand) approximately 2 ml over a timed collection period (2 min). The saliva samples were aliquoted into two separate sterile containers (LabServe, New Zealand) and stored at - 80°C until assay. Samples were analysed in duplicate using commercial kits (Salimetrics LLC, USA) and the manufacturers' guidelines. The minimum detection limit for the testosterone assay was 2 pg/ml with intra- and inter-assay coefficients of variation (CV) of 1.2 -12.7%. The cortisol assay had a detection limit of 0.3 ng/ml with intra- and inter-assay CV of 2.6 - 9.8%.

### Statistical Analyses

The accuracy of skill execution with sleep deprivation and treatments was examined using a two-way analysis of variance (ANOVA) with repeated measures on both the dominant and non-dominant passing sides. A two-way repeated measures ANOVA was also used to evaluate the effects of sleep state, treatments and any interactions for each hormonal variable. In addition, dominant versus non-dominant side skill performance during familiarisation trials and non-deprived performance versus familiarisation performance were examined similarly. The Tukey HSD test was used as the post hoc procedure where appropriate. Significance was set at an alpha level of p ≤ 0.05.

## Results

### Familiarisation training and dominant versus non-dominant passing side

A significant main effect for skill performance was identified over time [*F*(5, 108) = 38.44, p < 0.001]. Skill execution on both sides improved significantly (p < 0.001) across the first 5 sessions (Table 1) and then was unchanged between session 5 and 12. Variability within an individual on non-sleep deprived days was less than 5% and, between individuals in the group, was less than 15% and no significant differences were seen. A significant main effect was also identified for passing side [*F*(1, 108) = 53.85, p < 0.001] with dominant side skill execution found to be superior to the non-dominant side across all trials (p = 0.013). No interactions between passing side and time were found [*F*(5, 108) = 1.899, p = 0.1].

**Table 1:** Accuracy, out of 10 attempts (20 total per trial), for each of dominant and non-dominant passing sides on the first, fifth and twelve familiarisation trials.

	1^st ^Trial	5^th ^Trial ^a^	12^th ^Trial ^a^
Dominant	7.3 ± 0.8	9.0 ± 0.7	9.0 ± 0.4
Non-dominant ^b^	5.7 ± 0.8	8.3 ± 0.8	8.2 ± 0.7

Data presented as mean ± SD.

^a ^significantly different from the 1^st ^trial (p < 0.001), ^b ^significantly different from the dominant side (p = 0.013)

### Placebo non-sleep deprived versus familiarisation

Placebo administration on non-sleep deprived days did not produce a significantly different performance result to that seen in the last familiarisation trial [*F*(1, 36) = 0.00, p = 1.0], but a significant main effect was identified for passing side skill execution, this being consistently higher on the dominant side than the non-dominant side [*F*(1, 36) = 22.737, p < 0.001]. No significant interactions were identified for these variables [*F*(1, 36) = 0.00, p = 1.0].

### Placebo versus creatine or caffeine on dominant passing side

**Figure 1:**
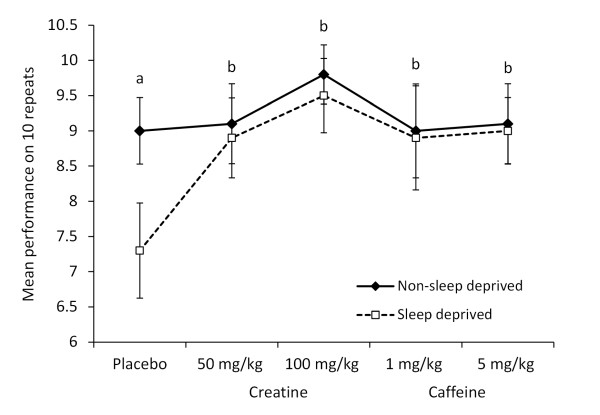
**Effects of sleep deprivation and acute supplementations on passing accuracy (dominant side)**. The mean ± SD is displayed for accuracy out of 10 passes on the dominant side (20 passes total per trial) for the 10 subjects under different treatment conditions (placebo; 1 or 5 mg/kg caffeine, 50 or 100 mg/kg creatine) either in non-sleep deprived or sleep deprived states. Dominant was chosen by the subjects as the side they believed showed better passing accuracy. All subjects completed 20 repetitions of the passing skill per trial, alternating passing sides (10 on dominant side). With placebo treatment sleep deprivation was associated with a significant fall in performance (a) (p < 0.001) compared to non-sleep deprivation. The 50 and 100 mg/kg creatine and 1 and 5 mg/kg caffeine doses were all associated with a significantly better performance (b) (p < 0.001) than the placebo conditions.

Repeated analyses revealed significant main effects for treatment condition [*F*(4, 90) = 19.303, p < 0.001], sleep state [*F*(1, 90) = 19.472, p < 0.001] and their interactions [*F*(4, 90) = 7.978, p < 0.001] on the dominant passing side (Figure 1). All of the caffeine and creatine doses produce a significant enhancement in skill performance when compared to placebo administration (p < 0.001). In the placebo condition, passing skill performance was found to be superior in the non-sleep deprived than the sleep deprived trial (p < 0.001).

### Placebo versus creatine or caffeine on non-dominant passing side

**Figure 2:**
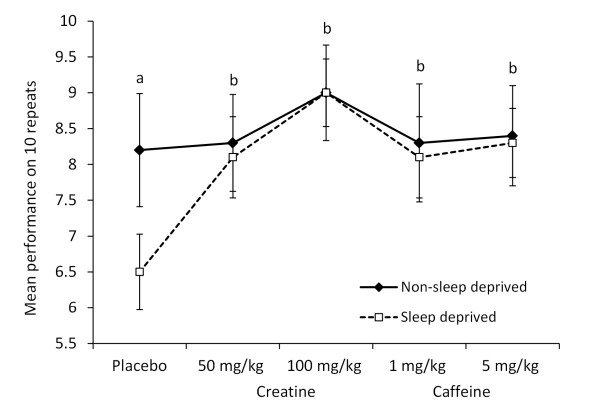
**Effects of sleep deprivation and acute supplementations on passing accuracy (non-dominant side)**. The mean ± SD is displayed for accuracy out of 10 passes on the non-dominant side (20 passes total per trial) for the 10 subjects under different treatment conditions (placebo; 1 or 5 mg/kg caffeine, 50 or 100 mg/kg creatine) either in non-sleep deprived or sleep deprived states. All subjects completed 20 repetitions of the passing skill per trial, alternating passing sides (10 non-dominant side). With placebo treatment sleep deprivation was associated with a significant fall in performance (a) (p < 0.001) compared to non-sleep deprivation. The 50 and 100 mg/kg creatine and 1 and 5 mg/kg caffeine doses were all associated with a significantly better performance (b) (p < 0.001) than the placebo conditions.

On the non-dominant passing side (Figure 2), significant main effects were identified for the treatment conditions [*F*(4, 90) = 14.871, p < 0.001], sleep state [*F*(1, 90) = 18.228, p < 0.001], and their interactions [*F*(4, 90) = 6.026, p < 0.001]. As with the dominant passing side, all of the caffeine and creatine doses produce a significant enhancement in skill performance from the placebo (p < 0.001) and, in the placebo condition, greater performance accuracy was noted in the non-sleep deprived (vs. sleep deprived) trial (p < 0.001).

Figures 1 and 2 summarise this data.

### Salivary testosterone and cortisol

**Figure 3:**
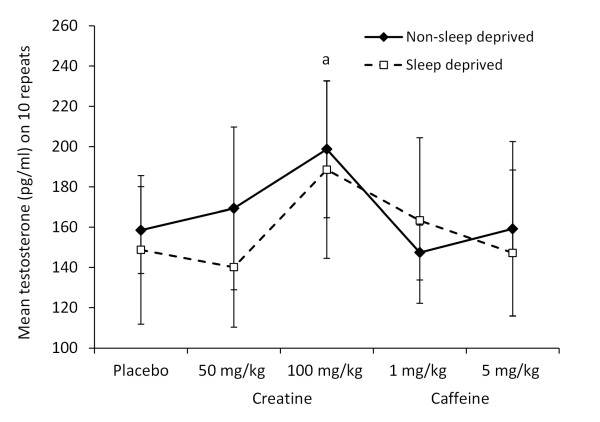
**Pre-trial salivary free testosterone (pg/ml) across treatments**. The mean ± SD is displayed for salivary testosterone under different treatment conditions (placebo; 1 or 5 mg/kg caffeine, 50 or 100 mg/kg creatine) either in non-sleep deprived or sleep deprived states. The 100 mg/kg creatine dose was associated with a higher concentration of testosterone (a) (p = 0.067) compared to the placebo treatment.

**Figure 4:**
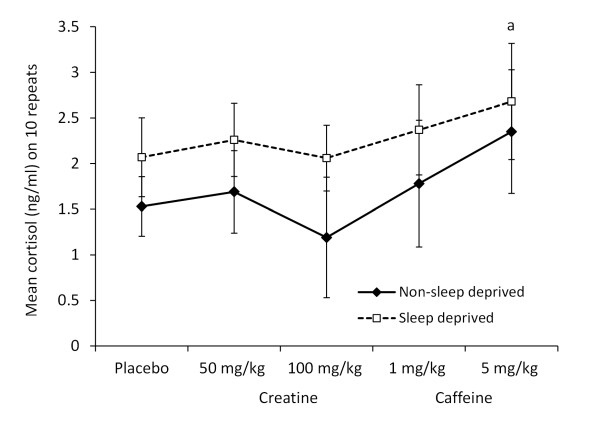
**Pre-trial salivary free cortisol (ng/ml) across treatments**. The mean ± SD is displayed for salivary cortisol under different treatment conditions (placebo; 1 or 5 mg/kg caffeine, 50 or 100 mg/kg creatine) either in non-sleep deprived or sleep deprived states. The 5 mg/kg caffeine dose was associated with a significantly higher concentration of cortisol (a) (p = 0.001) compared to the placebo treatment.

A significant main treatment effect [*F*(4, 90) = 4.855, p = 0.001] was identified for salivary testosterone (Figure 3), trending towards higher values after the 100 mg creatine dose (p = 0.067) than the placebo condition. There were no significant effects of sleep state [*F*(1, 90) = 1.602, p = 0.209], nor any interactions [*F*(4, 90) = 1.014, p = 0.405], on salivary testosterone. For salivary cortisol (Figure 4), significant results were noted for the main effects of treatment [*F*(4, 90) = 8.415, p < 0.001] and sleep state [*F*(1, 90) = 31.31, p < 0.001], but there were no interactions [*F*(4, 90) = 0.691, p = 0.6]. Cortisol was significantly higher with the 5 mg caffeine dose (p = 0.001) than the placebo treatment.

Figures 3 and 4 summarise this data.

## Discussion

Acute sleep deprivation is a common occurrence in the general population [23] including elite athletes. Such deprivation has been shown to affect some, but not all, physical and skill executions [15,20-22]. However, quantifying an effect in a team sport can be difficult. The repeated passing skill test we described herein is simple to perform, has sport-specific relevance and appears to be highly reliable across repeat testing. It is not however a one off, high-level performance task, rather a repeat of 20 fairly simple tasks, alternating passing sides. While we don't claim it to be in any way, yet, a valid performance measure it did reveal some interesting differences across acute sleep deprivation and across caffeine and creatine treatments.

In line with observations in other skill and psychomotor testing acute sleep deprivation reduced the accuracy over repeated trials. There was a general trend to a drop-off in accuracy latter in the repeats (second 10 of the 20 repeats). Whether this is a greater susceptibility to mental fatigue or not remains an interesting question, as does whether single skill repeats separated by more recovery time or by a similar physical activity with no real skill requirement would show a deficit in performance or not. In non-sport related psychomotor trials there is little evidence that a single episode of sleep deprivation produces significant deficit in a single task [15]; however across repeat tasks it is perceived that much greater effort is needed to maintain concentration [24].

Acute sleep deprivation has little effect on weightlifting performance [20], but can influence mood negatively [24] which may be a driving feature in mental performance changes. Caffeine, for example, has been shown to improve both mood and mental function following sleep deprivation [25]. It is not known how much mood and other cognitive function, particularly motivation on repeat skill tasks, interact. At the doses and administration time of caffeine use in this study we saw no evidence of an effect in non-sleep deprived subjects; however, there was a clear amelioration of skill performance deficit from the sleep-deprived trials with placebo administration. The psychostimulant effects of caffeine appear to be related to the pre and post synaptic brakes that adenosine imposes on dopaminergic neurotransmission by acting on different adenosine receptor heteromers [26], although numerous mechanisms are likely to be involved.

We did not see a dose related effect with caffeine supplementation, with 1 mg/kg and 5 mg/kg producing similar effects, nor did we see high individual variance (i.e. responders and non-responders). The absorption of caffeine in plasma following consumption has been estimated at between 30 and 90 min with half life of several hours [16], so the time between consumption and testing (90 min) in this study may have been too long to see all effects of differing caffeine dose, or any effect on non-sleep deprived performance. Nonetheless, at 90 min there was still clear evidence of a reduction in the effect of sleep deprivation on the skill measured and no evidence this was different between the 1 and 5 mg/kg dose.

Subjectively, a number of the subjects reported feeling slightly nauseous and anxious following the 5, but not 1, mg/kg administration of caffeine suggesting in other ways there were dose differences. Effective doses of caffeine (and their dose response nature) remain contentious in literature [1,5,6,27] possibly reflecting larger inter-subject variability in responses and different sensitivities of various physical and behavioural expressions. The subjects in this study were not regular caffeine users so arguably may have been more sensitive to lower doses than would be seen in more regular consumers. Certainly in the study herein 1 mg/kg was as effective as 5 mg/kg and from a practical perspective runs less risk of undesirable dose related side effects.

Chronic creatine supplementation has been shown to address certain aspects of sleep deprivation linked and other pathophysiology linked cognitive deficits [8,9,11,13,14,19], although very low dose chronic supplementation does not appear to improve function in non-sleep deprived healthy subjects [28]. Sleep deprivation is associated with a reduction in brain stores of phosphocreatine [10] and certainly in some disease states depletion of high energy phosphate stores has been measured, associated with cognitive deficit, and alleviated to some extent by creatine supplementation [13,14,29]. Interestingly, if there is an energy deficit associated with sleep deprivation then it seems logical to contend that repeat trials would be more susceptible than one off tasks. Our results and indeed other work on sleep deprivation do fit this pattern. If such depletion occurs and is acute, it also stands to reason that acute supplementation (as opposed to longer protocols) would address any associated deficit (given that brain uptake is not a time limiting factor). Little, if any, attention has been given to acute dosing with creatine, mainly because it is assumed that its effects come from a gradual build up of stores over time. We demonstrate here that an acute dose of creatine can ameliorate sleep deprived deficits in repeat skill performance trials. Again this possibly reflects the repeat nature of the trials and may not be observable in an acute one off mental skill performance.

Further in contrast to caffeine administration, the creatine dose of 100 mg/kg appeared to elicit a trend towards greater effect in skill performance than 50 mg/kg dosing, thereby suggesting potentially a dose dependent response. As in the case of caffeine we observed no individual variability suggestive of responders and non-responders or differential dose susceptibility, and no adverse effects were reported to us by the subjects. Clearly at the level of muscle function there does appear to be a division into responders and non-responders to longer term supplementation with different creatine protocols [4]. It is possible that this would be similar with longer term supplementation aimed at skill improvement, or alternatively brain-related creatine stores may operate slightly differently to muscle.

Acute sleep deprivation has been demonstrated in some studies to have small disruptive effects on basal hormonal concentrations [30,31]. Although salivary cortisol appeared to be elevated with sleep deprivation, this result did not reach statistical significance. Interestingly the higher dose of caffeine was associated with significant elevation in pre-trial cortisol, but not testosterone. High doses of caffeine have previously been demonstrated to acutely increase cortisol and, to a lesser extent, testosterone [20,32]. Whether such elevations have any significance in outcome is unknown. Cortisol is associated with arousal but also with anxiety [33]. Unfortunately we did not concurrently measure salivary alpha amylase in this study, which may also be a useful marker with respect to system arousal [34]. Testosterone was unaffected by sleep deprivation and by all treatments except the high dose of creatine, where there was a trend towards higher concentrations. We do not have useful speculation as to why this increase was seen, although it was across all subjects. Still, the increase was relatively small in magnitude and we doubt at this stage that it has any real physical or behavioural consequence. As we used saliva measures we cannot rule out some local oral cavity artefact effect of creatine. Free testosterone levels have, however, been linked to intra-individual variance in short timeframe muscular power [35], and long-term creatine supplementation has been reported as influencing testosterone metabolite pathways [36], so the observation is perhaps worthy of some follow-up.

Little has been published on acute creatine use as it has primarily been regarded as a longer term supplement to muscular function gain. In terms of brain and behavioural function it would appear it have some acute effects of value. It is also possible that the observed effects of caffeine and creatine reported in this and other studies are potentially summative and thus, would seem a logical progression for research.

## Conclusions

We observed a significant effect of acute sleep deprivation on performance (on both dominant and non-dominant passing sides) of a repeat simple skill test in elite rugby players. The deficit in performance with sleep deprivation was addressed by acute supplementation with either caffeine or creatine. In both cases, the two dosages tested had similar effects on skill performance. Both may offer practical and viable options prior to training and competition to assist skill performance when sleep loss has occurred.

## Competing interests

The authors declare that they have no competing interests.

## Authors' contributions

CJC participated in protocol design, conduct of the study, data analyses and manuscript preparation. LPK, CMG, SD and BC participated in protocol design, data analyses and manuscript preparation. All authors have read and approved the final manuscript.
